# Exercise-mediated IL-6 downstream effects modulate brain pathology–can exercise training protocols influence the downstream effects?

**DOI:** 10.3389/fneur.2025.1639427

**Published:** 2025-08-06

**Authors:** Hezhou Li, Anand Thirupathi

**Affiliations:** ^1^School of Physical Education and Health, Wenzhou University, Wenzhou, Zhejiang, China; ^2^Faculty of Sports Science, Ningbo University, Ningbo, China

**Keywords:** interleukin-6, physical exercise, brain, molecular signaling, neurodegeneration

## Abstract

The dual role of interleukin-6 (IL-6) as beneficial in physiological conditions and detrimental in pathological conditions has been a subject of research interest since its discovery. This has surpassed the traditional view of IL-6 as a pro-inflammatory cytokine, primarily due to its multifunctionality. To coordinate this multiple effect, several downstream signaling pathways are involved. Physical exercise mediates these downstream signals and accentuates the pleiotropic effects of IL-6 by enabling cross-talk between various organs, including muscles and the brain. In addition, IL-6 itself is a crucial signaling molecule that enhances exercise performance by maintaining muscle energy homeostasis. However, the specific mechanisms by which this molecule modulates overall brain physiology under different exercise conditions remain unclear. For example, chronic exercise with different exercise protocols could increase chronic plasma levels of IL-6, which could have an impact on brain health. Most studies in the literature have established the beneficial effects of exercise-mediated IL-6, but the impact of chronic elevation of IL-6 by exercise remains unclear. Additionally, the level of IL-6 determines the nature of molecular signaling that underlies all IL-6-mediated functions. This can be achieved by understanding both classic and IL-6 trans-signaling in different physio-pathological conditions. However, the mechanism by which exercise activates these two different classic and IL-6 trans-signaling pathways is less understood. Therefore, this review presents a comprehensive overview of how different exercises mediate IL-6-mediated benefits by discussing the full array of molecular signaling pathways.

## Introduction

The pleiotropic effects of cytokines arise from cross-reactivity and shared molecular signaling pathways, which may complicate the untangling of the molecular mechanisms of cytokines. Additionally, the perception of cytokines involved in disease progression as merely pro-inflammatory has shifted, as they play a crucial role in regulating metabolism, inflammation, and the immune response (1). However, the mechanisms by which cytokines execute these functions remain elusive in the realm of cytokine biology. Interleukin-6 (IL-6) is a crucial cytokine with multiple pleiotropic effects, enabling it to carry out a wide range of complex physiological functions. However, the activity of IL-6 may depend on the nature of IL-6 signaling (2). Mainly, three different signaling mechanisms, at least these conditions, are used to induce IL-6 response by the cells, termed classical IL-6R signaling that activates signaling pathways like Janus kinase/Signal transducer and activator of transcription 3 (JAK/STAT3) and phosphoinositide 3-kinases/protein kinase B (PI3K/AKT), IL-6 trans signaling, which induces a more sustained response of IL-6, and IL-6 trans presentation, which involves a cell–cell interaction (3). The discovery of IL-6 began in the late 1960s with the involvement of T-cells in antibody production (4, 5). In 1986, this T-cell-derived soluble factor, later renamed as B-cell stimulatory factor-2, became known as IL-6 (6, 7). Since then, this pleiotropic cytokine has maintained strong research interest due to its diverse biological roles (Table 1). Conversely, it has redundancy effects through its receptor-specific systems, called receptor-specific for IL-6 and gp130 (8).

**Table 1:** A complete chronological sequence of IL-6 response to exercise from its discovery to exercise-mediated targeted therapies.

Year of study	IL-6 response in biological samples	Exercise types	References
1991	IL-6 in blood	Bicycle exercise for 60 min (75% VO2max)	(120)
1999	IL-6 in blood	Endurance exercise with neuromuscular disease	(121)
1999	IL-6	Exercise on neuroendocrine response	(122)
2002	IL-6 in brain	Acute exercise on IL-6 release from brain	(16)
2004	IL-6 in knockout mice	Exercise mediated molecular signaling like AMPK mediate the IL-6 response	(123)
2005	IL-6 receptor	Acute bout of knee extensor exercise	(28)
2019	Tocilizumab with exercise	Exercise with tocilizumab effect on adipose tissue of obese	(124)

Following the discovery of IL-6, its role in brain cells, including astrocytes and glial cells, has been demonstrated in several studies involving various signaling pathways, such as the JAK/STAT, mitogen-activated protein kinase (MAPK), nuclear factor kappa-light-chain-enhancer of activated B cells (NFκB), PKC, Ca^2+^/calmodulin-dependent kinases, and the Prostaglandin E1 pathways (3, 9). However, all these signaling pathways act in both physiological and pathological conditions. Therefore, understanding their involvement in activating or deactivating IL-6 expression could provide a potential therapeutic target for treating brain diseases. Although pharmaceutical agents, such as antibodies and engineered fusion proteins, have been reported to block IL-6 in various models, including genetic knock-in strains and Drosophila (3, 9, 10), the mechanistic involvement of IL-6 signaling in diverse health and disease states remains poorly understood, especially in the brain. Therefore, elucidating the downstream targets of IL-6 could help to frame the contribution of IL-6 to brain health and diseases. Studies have reported that IL-6-mediated activation of STAT1 and STAT3 plays various pathological roles by triggering brain inflammation, which can promote brain apoptosis and lead to brain damage (11, 12). Nevertheless, it has also been implicated in neuronal survival (13). Therefore, designing a drug that simply targets the downstream signaling of IL-6 could also have deleterious effects in sensitive cells, such as those in the brain. In this context, physical exercise is one of the non-invasive remedies that can help sustained release of IL-6 via targeting its upstream signals like c-jun gene (14, 15). Additionally, chronic exercise may help regulate the IL-6 downstream targets without exaggerating the action of IL-6 (16); otherwise, it can also activate other downstream signaling pathways, such as PI3K/AKT, to induce neurodegeneration (17). This condition can be elucidated through resistance exercise, which modifies STAT3 signaling in the brain by regulating IL-6 levels to facilitate neuroprotection (16). This may be the mechanism through which exercise mediates the binding of STAT3 to brain-derived neurotrophic factor (BDNF) and protein Interacting with C Kinase - 1 (PICK1), thereby enhancing neural plasticity via the IL-6 response (18). However, high-intensity exercise disrupts the IL-6 response and the redox balance, which adversely affects insulin signaling in the brain via the PI3K/AKT pathway (19–21). Other signaling molecules, such as protein kinase C (PKC), are known to be increased by exercise in response to interleukin-6 (IL-6), which induces neuroinflammation, oxidative stress, and apoptosis (22). Conversely, PKC regulates neuronal activity and survival by activating prostaglandin E_2_ (PGE2) through the EP4 receptor (23); exercise is also known to regulate PGE2 (24). However, the mechanisms by which different exercise protocols mediate this effect via IL-6 in improving neuronal survival are unknown. Exercise types, such as aerobic exercise, may also inhibit the IL-6-mediated STAT3 signaling pathway by blocking the JAK2/STAT3 pathway, thereby enhancing cognitive function and neuroprotection (25). However, it is important to elucidate the involvement of other antiinflammatory cytokines to ensure the IL-6-mediated benefits. Additionally, the effect of exercise protocols, such as intensity, duration, and type, on the IL-6 response to activate IL-6-mediated downstream targets is poorly understood. Therefore, this review aims to discuss the potential downstream targets of IL-6 for maintaining brain health during exercise.

## How do the downstream effects of IL-6 signaling mediate the brain physiopathology- role of exercise

**Figure 1:**
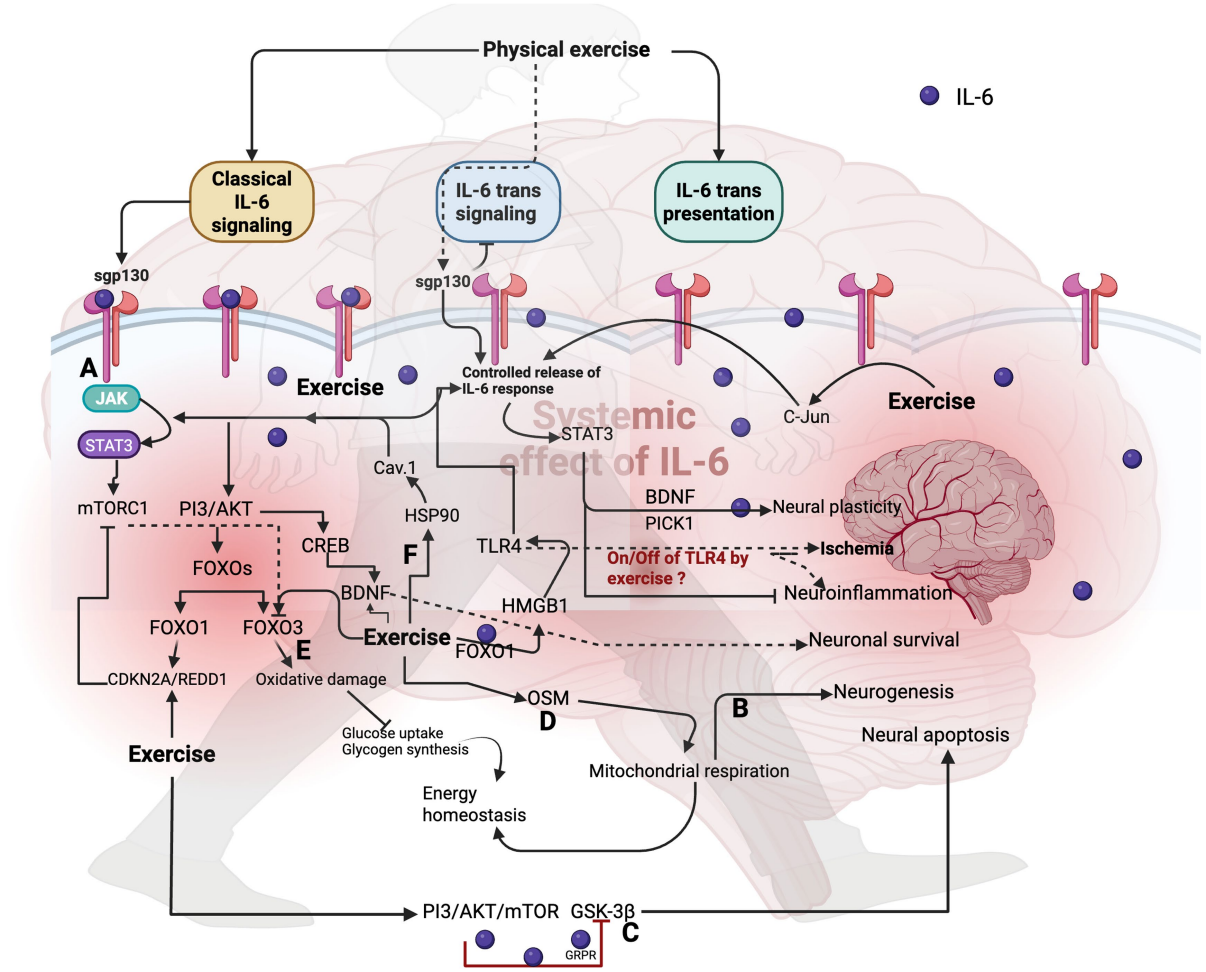
Effect of physical exercise on IL-6 signaling in brain. **(A)** Physical exercise increases the sgp130 to decrease IL-6 trans-signaling, thereby inducing sustained release of IL-6 in the brain to activate JAK/STAT3, which in turn triggers BDNF and PICK1 to increase neural plasticity, neuronal survival, and neurogenesis. Additionally, exercise targets the c-Jun protein to regulate the IL-6 response in the brain. **(B)** Exercise-induced classical signaling of IL-6 activates its downstream targets, such as JAK/STAT3, to improve neurogenesis. **(C)** Exercise-induced IL-6 inhibits GSK-3β through the PI3K/AKT/mTOR pathways to decrease neural apoptosis via the GRPR. Additionally, exercise-mediated IL-6 triggers FOXO1 signaling, which in turn activates TLR4 to increase IL-6 production. However, overexpression of this may induce ischemia and inflammation in the brain, and the role of shutting down these molecules under exercised conditions in preventing ischemia and neuroinflammation is unknown. **(D)** Exercise-induced OSM enhances mitochondrial respiration, thereby improving energy homeostasis in the brain. **(E)** PI3/AKT/FOXO3-induced oxidative damage in the brain is prevented by the exercise-mediated IL-6 response by CDKN2A/REDD1, which targets mTORC1 and FOXOs. **(F)** Exercise influences the expression of heat-shock protein 90 (HSP-90) and caveolin-1, thereby activating the STATs pathways, which is mediated by exercise-triggered IL-6.

Depending on the stress response employed by the cells, IL-6 can be defined as an adipokine, myokine, monokine, or neurotrophic factor (26). This response may be triggered by exercise, infection, trauma, or specific disease conditions, such as cancer or autoimmunity. As mentioned, exercise regulates the IL-6 signaling mechanisms to elicit the controlled response of IL-6 within the muscle and to other organs (26). For example, exercise primarily utilizes the classical IL-6R signaling pathway to activate IL-6-mediated downstream signaling, thereby enhancing performance by maintaining energy homeostasis and providing anti-inflammatory benefits associated with exercise (26, 27). Mainly, the exercise-induced local effects of IL-6 lead to the systemic effects via mbIL-6R in skeletal muscle, mediated by 5’ AMP-activated protein kinase (AMPK) activation, as evidenced by the increased expression of mbIL-6R in the myocytes of resistance-trained individuals (27, 28). Thus, exercise-mediated IL-6 classical signaling may induce systemic effects in the brain by facilitating the interaction of IL-6/gp130 to target its downstream signaling, such as JAK/STAT (27, 28) (Figure 1).

The increase in IL-6 levels is associated with age-related pathological consequences. For instance, a study found that IL-6 concentration increased from 1.4 pg./mL in the 65–74 year age group to 3.5 pg./mL in those aged 85 years among men (29). Similarly, women showed an increase in IL-6 levels from 1.1 to 2.1 pg./mL over the same age range (29). Importantly, this rise in IL-6 is independent of residual confounding factors that influence the development of age-related neurodegenerative diseases (30). One possible explanation for this increase is that elevated reactive oxygen species (ROS) production or reduced clearance of ROS may stimulate IL-6 production (29). This, in turn, can worsen IL-6-mediated pathological effects through the IL-6/JAK/STAT3 signaling pathway, which is a significant driver of glioblastoma. While IL-6 activates STAT3 and can offer neuroprotective effects, both the neuroprotective and oncogenic impacts of IL-6 depend on the dosage and duration of its presence. For example, low levels of IL-6 are beneficial for neuronal survival, while elevated or prolonged IL-6 exposure can promote tumor growth by activating STAT3 signaling (31). Moreover, the activation of the IL-6/STAT3 pathway influences downstream targets that can either induce neuroprotection or contribute to oncogenic effects. For example, IL-6/STAT3 triggers the activation of BDNF and TrkB, which in turn enhances STAT3 activation, thereby improving BDNF expression through a positive feedback loop mechanism that promotes neuroprotection (32). Whereas IL-6/STAT3 promotes the Bcl-XL, Mcl-1, and VEGF, to inhibit apoptosis and promote cell survival by increasing angiogenesis and blood supply to the tumor (33). Additionally, the tumor microenvironment plays a crucial role in triggering the pro-tumorigenic effects of IL-6 and STAT3 (34).

Exercise can also influence the upregulation of sgp130, leading to the inhibition of IL-6 trans-signaling (27). This can decrease the sickness behavior induced by LPS, improve brain recovery from injury, cognitive functions, and reduce anxiety by inhibiting IL-6 trans-signaling in the murine microglia cell line and mouse model (35, 36). Downstream targets of IL-6, mainly JAK/STAT, regulate neuroinflammation and affect the survival of neuronal and glial cells, as evidenced by the dysregulation of JAK/STAT pathway in brain disorders, such as brain cancers, ischemia, and Alzheimer’s disease (AD). Exercise mitigates this condition by targeting the membrane-proximal region of the receptors to interrupt the interaction of IL-6-mediated JAK/STAT activation (37). For example, resistance exercise for 12 weeks transiently increases IL-6 and STAT3, facilitating adaptive responses to high-intensity exercise (37). However, no studies in the literature have discussed how exercise affects this highly conserved membrane-proximal region of the receptors, thereby interrupting the IL-6-mediated JAK/STAT signaling in brain diseases.

Additionally, IL-6 trans-signaling alters the toll-like receptor 4 (TLR4)-dependent signaling in the gp130 (F/F) knock-in mutant mice to modulate the inflammatory response through STAT3 signaling (38). Treadmill exercise reduces TLR4 overexpression in cerebral ischemia, thereby lowering IL-6 secretion through the HMGB1/TLR4-mediated mechanism (39, 40). Additionally, exercise influences the expression of heat-shock protein 90 (HSP-90) and caveolin-1, thereby activating the STATs pathways, which is mediated by exercise-triggered IL-6 (41, 42). For instance, treadmill exercise for 28 days increases the caveolin-1 and vascular endothelial growth factor (VEGF) signaling to promote neurogenesis in the ischemic rats, possibly through an exercise-mediated IL-6 mechanism (41, 42). However, caveolin-1 upregulation inhibits the STAT3 signaling in brain metastasis (43), and further research is required to determine whether IL-6 shuts off STAT3 signaling to activate caveolin-1-mediated benefits in the brain upon exercise. It is known that IL-6 activates HSP90 to suppress protein aggregation and facilitate Aβ clearance in AD conditions (44). Additionally, an acute bout of exercise activates HSP90 by upregulating IL-6 (45). Oncostatin M (OSM) is associated with muscle atrophy, and the muscle-specific deletion of OSMR may prevent muscle atrophy via the IL-6-mediated JAK/STAT3 pathway (46). In the context of brain physiology, OSM disrupts the blood–brain barrier function by activating the JAK/STAT3 signaling pathway *in vitro* (47). Conversely, OSMR regulates the brain tumor stem cells’ proliferation via interacting with NADH ubiquinone, NADH ubiquinone oxidoreductase 1/2 (NDUFS1/2) of complex I to promote mitochondrial respiration, suggesting the dual role of this OSMR (48). However, the involvement of IL-6 in this context upon exercise requires further exploration. IL-6 activates other signaling pathways, such as the PI3K/AKT/the mammalian target of rapamycin (mTOR) glycogen synthase kinase-3 beta (GSK-3β) and gastrin-releasing peptide (GRP) receptor (GRPR) pathways, in hippocampal neurons of mice with autism spectrum disorder (49). It has been reported that exercise regulates these signaling to improve brain health. For example, the PI3K/AKT signaling pathway is crucial for brain function by activating its downstream targets, including GSK-3β, mTORc, FOXOs, and CREB (50). Treadmill exercise for 30 min increased the expression of PI3K/AKT proteins in the rat’s brain by inhibiting GSK-3β, thereby improving depression (51). However, this study did not investigate whether IL-6 mediated this response to improve depression symptoms. Studies have reported that the elevation of GSK-3β is associated with the loss of dopaminergic neurons, an increase in amyloid beta production, and the formation of neurofibrillary tangles (52, 53). The inhibition of GSK-3β via targeting the IL-6-mediated PI3/AKT/mTOR pathway decreases neural apoptosis and affects autism spectrum disorder via GRPR (52, 53). Exercise-induced mTOR activation improved cognition and emotional behavior in mice models with IL-6 overexpression (26, 54, 55). Studies have reported that mTOR regulates protein synthesis and degradation in the context of Alzheimer’s disease (AD) pathogenesis (56).

Moreover, activation of the PI3K/AKT pathway via IL-6 triggers FOXO1 signaling, which can further activate TLR4, thereby increasing the expression of IL-6; this vicious cycle may be involved in regulating the inflammatory process in the brain (57). For instance, aerobic exercise downregulates FOXO1 to reduce neural apoptosis in the hippocampus region through the CDKN2A/REDD1 pathway, and this may occur via exercise-mediated activation of death-associated protein kinase 1 (58). In addition, exercise-induced IL-6 activates CREB via the PI3K/AKT pathway to decrease depressive disorder, primarily by activating BDNF signaling in the hippocampus (59). This entire downstream target is manipulated by exercise-activated IL-6, without the release of other proinflammatory cytokines; however, it may be linked to lactate formation during exercise (60). Conversely, exercise-induced IL-6 mediates the PI3K/AKT pathway, which may activate an anti-inflammatory response to reduce oxidative stress and neuronal damage by targeting FOXO3 and NF-κB (61). Additionally, this condition enhances insulin sensitivity, facilitating increased glucose uptake by neurons and glycogen synthesis to maintain energy homeostasis in brain cells (62). The 2IL-6, ROS, and NF-κB play a crucial role in neurodegeneration by creating a positive feedback loop that triggers inflammation and neuronal damage. For instance, reduced levels of IL-6 and NF-κB have been shown to decrease neuroinflammation induced by LPS in BV2 cells (63). Additionally, a study indicated that sulforaphane, known for its antioxidant properties and ability to reduce ROS, lowers IL-6 and NF-κB levels in a rat model of AD, suggesting that IL-6-induced ROS activation triggers the NF-κB and vice versa (64). Furthermore, chronic treadmill exercise for 12 weeks, five days a week, mitigated IL-6 and NF-κB levels in an AD mouse model, likely by reducing ROS formation through the modulation of IL-6 and NF-κB (65). Therefore, targeting this pathway may provide therapeutic value for neurodegenerative diseases.

The role of IL-6 in targeting BDNF responses for neuroregeneration and the treatment of depressive disorders is critical, particularly as exercise is associated with elevated BDNF levels (66). For instance, unaccustomed exercise has been observed to reduce BDNF responses, with this decrease persisting for at least 24 h following the unaccustomed stretch-shortening activity (67). Notably, these changes do not correlate with IL-6 levels, indicating that specific exercise modalities may regulate BDNF responses independently of IL-6 activation (67). Moreover, the activation of CREB by CRTC1 has been linked to improvements in mood disorders (68), with evidence that CREB can bind to the promoter region of the IL-6 gene, thus influencing IL-6 transcription. While exercise is known to enhance CREB activation, physiological hypertrophy resulting from exercise may also impact this activation (69), potentially affecting IL-6 levels and reversing inflammatory responses in neurodegenerative conditions. Stress-induced proteins, such as Regulated in Development and DNA Damage-Response 1 (REDD1), are associated with metabolic disorders and neurodegeneration, while also contributing to oxidative stress and autophagy (70). This may be dependent or independent of mTORC1, but IL-6 may be crucial for activating REDD1 by carrying out mTOR-dependent or its other downstream target STAT3 to regulate REDD1 (70, 71). In this case, exercise-mediated hypoxia could activate REDD1, either triggering IL-6 or hypoxia induced by exercise, to increase REDD1 without IL-6 response in the brain (72). Treadmill exercise increased the expression of REDD1 via decreasing mTORC1 in the rats (72). Receptor-interacting protein kinase 1 (RIPK1) regulates neural apoptosis (73). Treadmill exercise modulates RIPK1 to regulate the IL-6-mediated response via MAP3K5/JNK and NF-κB, thereby decreasing neuroinflammation in the hippocampus of aged mice (73).

## How exercise triggers the of IL-6 response for altering brain functions?

**Figure 2:**
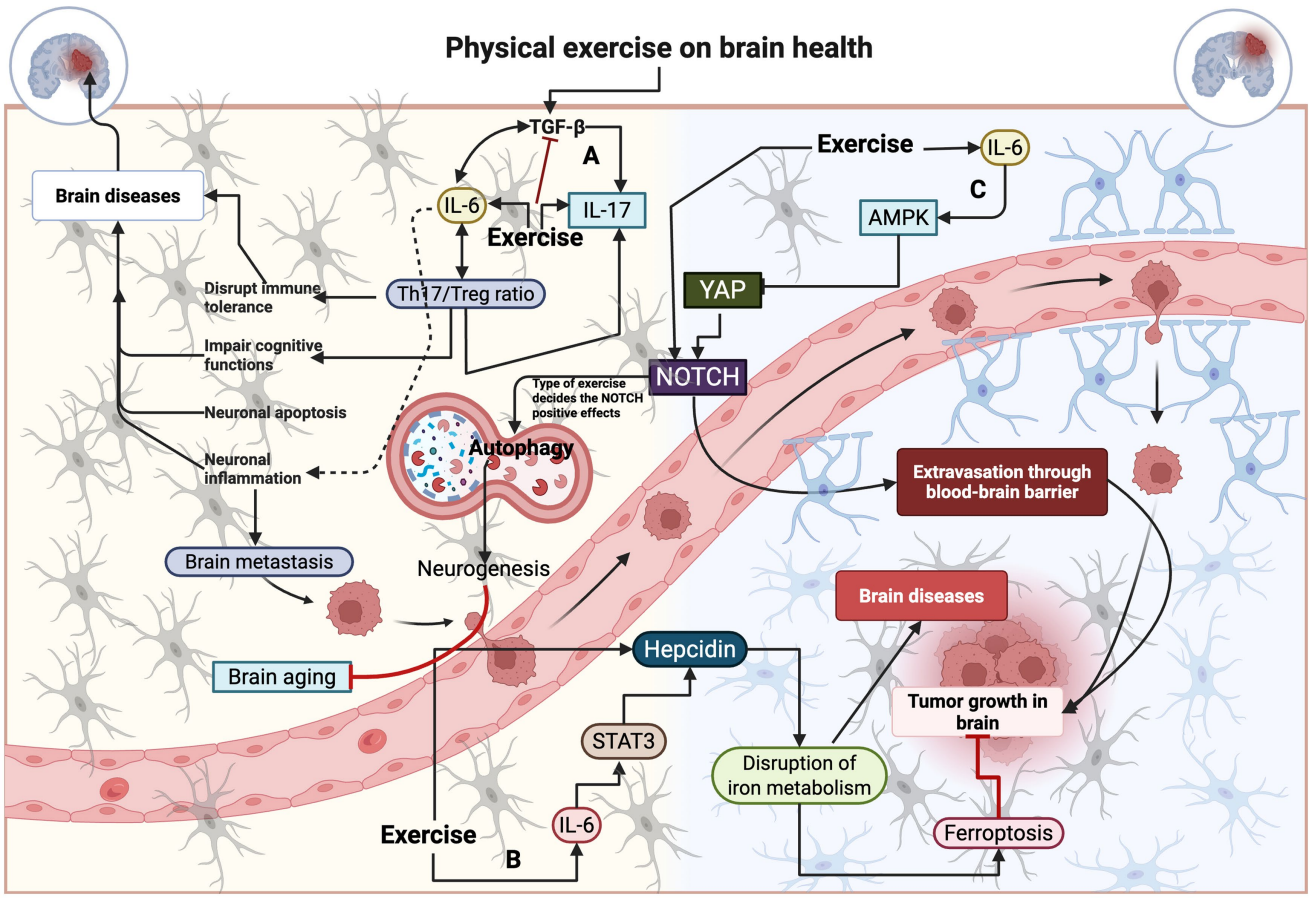
Effect of exercise on mediating pleiotrophic functions of IL-6 in the brain. **(A)** Physical exercise induces TGF-beta, which in turn stimulates IL-6, thereby regulating the ratio of TH17 to Treg cells, while also increasing IL-17 to enhance brain function. **(B)** Exercise-mediated IL-6 response alters the iron metabolism via targeting hepcidin. **(C)** Energy demand under exercised conditions activates the AMPK and YAP to induce the positive effect of NOTCH, which can induce neurogenesis in the brain by activating autophagy and preventing brain aging.

The prompt synthesis of IL-6 could carry pleiotropic effects by modulating various cytokines and growth factors. Physical exercise can accelerate this process. For example, prolonged exercise induces the release of IL-6 from skeletal muscle, which is then released into circulation to modify the secretion of other proteins, such as TLR-4, thereby regulating physiological processes in the brain, including neurogenesis (74). However, these two molecules can also play a role in inducing an inflammatory response that leads to neuronal damage, and the role of physical exercise in this condition is ambiguous. Transforming growth factor-*β* (TGF-β) contributes to AD pathogenesis during brain injury, in combination with IL-6, by promoting the expression of ROR-γt to regulate IL-17 (75). This elicits anxiety and affects social behavior (76). However, exercise can reverse this condition by increasing adaptive signaling, including PGC-1α, mTOR, and AMPK, without altering the IL-6-mediated benefits (27). For example, recreationally active individuals have reduced levels of sIL-6R, which favors adaptive signaling to exercise, resulting in a balance shift toward higher classical signaling and less trans-signaling, thereby avoiding a pro-inflammatory response to the exercise program (27). In addition, combined training decreased the expression of IL-17 in the plasma of multiple sclerosis patients (77), which could reciprocally decrease the expression of TGF-*β* without triggering IL-6 (78). In addition, elevated levels of IL-6 are linked to increased levels of Th17/Treg ratio, which disrupts the immune tolerance in the brain cells and causes neurological disorders (79) (Figure 2). The Th17/Treg ratio imbalance exacerbates cognitive impairment by modulating STAT3 activation in mice, potentially illustrating the immune regulatory effects of STAT3 through IL-6-mediated Th17/Treg levels (80). Treadmill exercise for 4 weeks (5 days/week) in adult ischemic rats regulates the ratio of Th17/Treg by decreasing neuronal apoptosis via inhibition of the IL-6-mediated signaling pathway, such as JAK2/STAT3 (81).

Next, IL-6 contributes to the development of T follicular helper cells (Tfh), which can be linked to the pathophysiology of neuromyelitis optica spectrum disorders (82). Karnowski et al. reported that Tfh cells were reduced in IL-6 knockout mice with the absence of IL-21, which is linked to the driving of neuroinflammation and fat accumulation in microglia (83). However, specific condition for exercise-mediated IL-6-induced Tfh development need to be reported, along with the corresponding mechanisms. IL-6 induced recruitment of gp130 triggers the yes-associated protein 1 (YAP) signaling and neurogenic locus notch homolog protein (NOTCH) signaling to promote glioblastoma growth in the human brain (84, 85). Exercise has been reported to regulate the YAP, especially in energy-deprived conditions, via AMPK, which phosphorylates the YAP (86). Additionally, various types of exercise activate the NOTCH signaling pathway, thereby decreasing brain aging by promoting autophagy and hippocampal neurogenesis, which in turn improves spatial learning and memory in rats (87). Nevertheless, the role of the IL-6 mechanism in this condition needs to be established. IL-6 also has metabolic controls in the brain by regulating glucose concentration in HFD mice (88). Mainly, IL-6 trans-signaling regulates systemic glucose homeostasis in hypothalamic neurons (89). IL-6 regulates energy expenditure in the hypothalamic region, as evidenced by the modulation of fat metabolism in obese conditions (90). Studies have shown that schizophrenia disorder is linked to altered glucose and fat metabolism, and the infusion of IL-6 in IL-6 knock-out mice failed to induce schizophrenia disorder (91, 92), indicating that IL-6 elevation could be a general feature of major depressive disorders like schizophrenia. However, the precise mechanism by which IL-6 is linked to these disorders remains elusive. Due to its diverse roles as both pro-inflammatory and anti-inflammatory mediator in the brain, IL-6 can contribute to a range of outcomes from neuroprotection to neurodegeneration. These effects likely stem from complex molecular interactions within the central nervous system, as well as the actively working skeletal muscle, which is primarily influenced by the cellular environment, blood–brain barrier permeability, and the specific IL-6 signaling pathways that are activated during exercise. Exercise elevates the circulating IL-6, indicating that working skeletal muscle is the predominant source of IL-6. However, astrocytes are another major source of IL-6, especially in conditions such as brain injury, hypoxia, and inflammation. This suggests that muscle-derived IL-6 is generally considered beneficial during exercise, while brain-derived IL-6 has both beneficial and detrimental effects, which can potentially contribute to increased neurodegeneration (93). Moreover, the IL-6-mediated metabolic boost can be utilized by the exercised brain to enhance energy homeostasis (16), while also ensuring a constant supply of glucose and fats to the working muscles (94). Moreover, IL-6 plays a crucial role in brain iron metabolism, as evidenced by the decrease in serum iron and the increase in IL-6 levels in the serum (95). Sterling et al. showed that IL-6 induces iron sequestration in neurons in response to pathological alpha-synuclein in both humans and mice with PD (96). Regular running exercise triggered the redistribution of iron in altered brain iron metabolism by decreasing cortical hepcidin levels, coupled with increased IL-6 levels in the cortex and plasma, in an AD mouse model, possibly through an IL-6/STAT3/JAK1 pathway-mediated mechanism (97).

## Therapeutic possibilities of IL-6- role of exercise

The level of IL-6 obviously contributes to the pathogenesis of brain diseases. Regular physical exercise can decrease IL-6, while also helping to block downstream signalings of IL-6. For example, the increase in IL-6 in cerebrospinal fluid affects neuroplasticity in MS patients, and physical exercise can inhibit this condition by regulating IL-6 (98–100). Next, targeting IL-6 signaling, such as STAT3, could alleviate memory impairment and glucose intolerance in AD, and exercise may serve as a STAT3 inhibitor to improve this condition (20). A study has shown that exercise, such as resistance training, decreases the STAT3 signaling in muscle atrophy (20). Whereas running exercise improves STAT3 signaling by targeting GPC6 to elicit neuroprotection in a mouse model of cerebral artery occlusion (101), indicating that each exercise protocol elicits different effects to target the IL-6-mediated signaling. Designing an exercise strategy that targets gp130-mediated signaling could have therapeutic application in brain tumors like glioblastoma by influencing endothelial-mediated actions (102), and decreasing neuronal loss and hyperinflammation (103), evidenced by the increase of gp130 by running exercise, which suppresses the STAT3 signaling to improve mitochondrial quality (104). Moreover, finding the association between exercise training and different protocols on single-nucleotide polymorphisms (SNPs), such as rs2228145 SNP, could modify the expression of sIl-6R, which might extend the half-life of IL-6 from minutes to hours; thus, it can transiently increase its effects from its production site to distant regions of the brain to produce systemic effects to target neurodegenerative diseases (27).

Engaging in 30 min of aerobic treadmill exercise per day, starting at an initial running speed of 2 m/min and gradually increasing to 8 m/min for 20 min, can activate the PI3K/AKT pathway. This activity may inhibit GSK-3β through a potential increase in IL-6, which in turn can decrease neurofibrillary tangles by hyperphosphorylating tau protein (55, 105). Additionally, treadmill exercise influences the IL-6-induced inhibition of mTOR via AMPK without activating STAT signaling (55). This mechanism is crucial in driving the pathogenesis of AD (105). In contrast, 12 weeks of resistance training—conducted 3 days a week with a 48-h rest interval between sessions—was shown to increase IL-6 levels with aging, thereby exacerbating the pro-inflammatory response (106). However, this response can be reversed with regular exercise training (106). Studies have indicated that both aerobic and resistance exercises affect the circulation of IL-6 by increasing levels of irisin (107, 108). For instance, cycling exercise performed three times a week for 55 min at 70% VO2 max enhanced the irisin response (108), while running three times a week for 60 min also increased irisin levels (107). Furthermore, resistance training for 12 weeks, twice a week for 55 min, resulted in an increased irisin response (107). Although these studies did not specifically assess IL-6, a study demonstrated that the release of irisin reduces IL-6 in astrocytes, decreases the expression of COX-2, and inhibits the phosphorylation of AKT by blocking NFκB activation (109). This process helps to protect neurons from Aβ toxicity in AD (109), suggesting that the exercise-induced hormone irisin plays a crucial role in regulating the IL-6-mediated response in neurodegenerative diseases. Activities such as walking, running, cycling, or swimming can increase the release of IL-6 and other anti-inflammatory cytokines, thereby improving insulin sensitivity (137). Insulin resistance is a hallmark of various neurodegenerative diseases, such as AD and PD. Additionally, resistance training enhances muscle strength, contributing to overall health and mobility in individuals with different neurodegenerative conditions. Therefore, performing both aerobic exercise (3 to 5 days per week, 30–40 min at 60–80% intensity) and resistance training (2–3 days per week) can improve outcomes for individuals with neurodegenerative conditions (110) (Table 2). However, personalized interventions should be tailored to individual circumstances.

**Table 2:** Aerobic versus resistance training protocols and their effects on IL-6 response in neurodegenerative conditions.

Aerobic exercise	Molecular target for IL-6 mediated pathophysiological in brain	References	Resistance exercise	Molecular target for IL-6 mediated pathophysiological action in brain	References
Voluntary running wheel exercise (mice)	Aerobic training decrease hippocampal inflammation and neurodegeneration by reducing IL-6	(125)	Resistance exercise program is followed three sets of four lower limb exercises, (1) leg press, (2) leg curl, (3) leg extension, and (4) calf raises for 12 weeks	IL-6 level was increased in mild cognitive impairment condition.	(126)
Walking exercise for 5 min over ground on 15 feet long in MS people	Faster walking speed increases the BDNF/IL-6 ratio for triggering repair phenotype	(99)	Resistance training was followed in aged rats with a maximal load test	IL-6 was decreased when compared to aerobic training in the cortical and hippocampal region.	(127)
Motor treadmill at a speed of 10 m/min for 90 min for 2 weeks in mice	Physical exercise decrease the IL-6 expression via irisin to elicit neuroprotection	(128)	Resistance exercise as climbing ladder exercise intervention was followed for 8 weeks	IL-6, iNOS and STAT3/STAT3 levels were increased for improving spatial learning and memory and decrease the neuronal damage, neuroinflammation by promoting polarization of M2 microglia in the hippocampus.	(129)
7 days of treadmill exercise adaptation program is followed, then; 15 min of exercise at the speed of 5 m/min on day 1 and 2 then 8 m/min on 3 and 4 and 12 m/min on 5 to 7 days. Then formal exercise protocol is followed for 5/week for 30 min in mice	Exercise decreased the IL-6 for reducing neuroinflammation in mice with AD, by affecting the SUMO1 and IGF1R	(87)	Resistance exercise was performed 5 times/week for 10 weeks in mice	IL-6 was increased to enhance the COX2 expression in the cortex and hippocampus, which is independent of systemic inflammatory process.	(130)
Animals spent 3 h in running wheels in AD mouse model.	Improves the neurogenesis via promoting the levels of IL-6/BDNF via FNDC5.	(131)	Resistance training was performed for 5 times/week over 4 weeks. RE protocol is consisted of climbing a ladder with a progressive load.	Resistance training restored the IL-6 level to decrease amyloid load, and. Inflammation in AD model.	(132)
treadmill running exercise for 3 days	IL-6 is decreased, which in turn to switch microglia polarization, thus improving motor function after stroke via MMP12	(129)			

## Does exercise duration and intensity influence the IL-6 response?

Although exercise is strongly associated with IL-6 response, the duration and intensity of exercise as contributing factors in determining the IL-6 response, especially in brain physiology, are unknown. Some studies have shown that a longer duration with moderate intensity significantly increases the IL-6 response within skeletal muscle and its surrounding interstitial fluid, thereby eliciting its pleiotropic effects on the brain (16, 111). This is evidenced by a greater increase in IL-6, approximately 8,000-fold, after a foot race of up to 246 km (111). In addition, prolonged exercise induces the release of IL-6 from the brain, evidenced by the no release of IL-6 from brain after 15 min of exercise, while 60 min the same exercise induced the small release of IL-6 and this was increased upto fivefold after the second bout of exercise in the brain (16). Moreover, exercise intensity is another factor that greatly elevates the IL-6 response (112). For example, cycling exercise at low intensity for 35 min increased the IL-6 response by up to 1.4-fold, whereas the same exercise at high intensity elevated the IL-6 response by up to 2.7-fold (27). However, all these studies are used aerobic training and the role of resistance exercise with higher intensity on IL-6 response is limited when compared to aerobic exercise, and exploring future research on resistance training on the brain functions, especially with neurodegenerative populations such as AD, PD and dementia could effectively integrate the types of exercise, which improve the functional outcomes of those people. Other factors, such as glycogen availability and the training status of the individuals, as well as the mode of exercise in relation to intensity and duration, also influence the IL-6 response. The possible mechanism that involves increasing the IL-6 response using these factors may be mediated by AMPK signaling that elevates IL-6 under a low-energy state and vice versa. Another mechanism involves increasing calcium signaling through exercise, particularly chronic exercise that can induce the IL-6 response, thereby regulating iron efflux (113, 114). This mechanism also decreases exercise-induced fatigue by astrocytes (115).

## Why do exercise protocols matter for IL-6 signaling in AD/PD?

Exercise protocols play a crucial role in maintaining IL-6 signaling in AD and PD by modifying the inflammatory process (133–135). These protocols affect various signaling pathways, which can help slow disease progression. For instance, acute exercise increases the IL-6 response and elevates anti-inflammatory factors such as IL-1RA and IL-10. These factors, in turn, downregulate pro-inflammatory cytokines, including TNF-*α* and IL-1β, in individuals with AD (116). On the other hand, chronic exercise reduces the elevated levels of IL-6, TNF-α, iNOS, and COX-2, thereby alleviating neuroinflammation in AD (65). Regular exercise is also beneficial for maintaining brain volume in the elderly, which can help prevent AD and dementia (136). The intensity and frequency of exercise can significantly influence both the magnitude and duration of IL-6 release. Additionally, the type of exercise performed can affect the release of IL-6 and help mitigate the pro-inflammatory response. For example, resistance-type exercise has been shown to decrease the activation of NF-kB in mice with PD by lowering IL-6 levels (117). This reduction can improve short-term memory, and the effects of resistance exercise are comparable to levodopa treatment in PD (117). Moreover, an increase in IL-6 is correlated with poor physical functioning, weakness, and fatigue in PD patients, likely due to heightened muscle catabolism that contributes to sarcopenia (118). Additionally, a single bout of resistance exercise performed three times a week for 12 weeks can enhance the IL-6 response, activating downstream targets such as STAT3, c-MYC, c-FOS, and SOCS3 (37, 119), which are associated with the aging process. Therefore, carefully selecting and tailoring exercise protocols may help minimize the detrimental effects of IL-6 signaling while harnessing its beneficial effects to enhance outcomes for individuals with AD and PD.

## Limitations

Prescribing exercise to individuals with specific neurodegenerative conditions presents several limitations and challenges. For example, in conditions like PD, the progressive loss of motor function can make it difficult for individuals to perform even basic exercises as the disease advances. Additionally, cognitive impairments in individuals with AD can hinder their ability to understand and follow exercise programs. The benefits of exercise and the tolerance for it can vary widely among individuals with different neurodegenerative conditions. Furthermore, there is a lack of established exercise protocols that have been proven safe and effective in alleviating symptoms of neurodegenerative diseases. The feasibility of patients engaging in exercise often depends on the specific disease condition, its severity, and the individual’s overall health status. Comorbidities associated with neurodegenerative diseases, such as hypertension and osteoarthritis, can also significantly impact exercise performance. Individuals with these comorbidities may have decreased exercise tolerance. Moreover, genetic factors like IL-6 polymorphisms (for instance, rs2228145) can influence an individual’s response to exercise by altering the risk of disease progression through changes in IL-6 signaling. Therefore, understanding the relationship between IL-6 and rs2228145 could greatly assist clinicians and exercise professionals in personalizing exercise programs for individuals with neurodegenerative diseases. A multidisciplinary approach that includes physical therapy, neuropsychological rehabilitation, or occupational therapy can further optimize exercise programs and improve patient outcomes.

## Conclusion

The pleiotropic effects of IL-6 impede the development of strategies that can target the IL-6 response in brain diseases. Evidence suggests that inhibiting the downstream targets of IL-6, such as JAK/STAT3 and PI3K/AKT, may mitigate the normal physiological functions of the brain, as these pathways also play roles in enhancing physiological functions. Therefore, careful attention is necessary before designing potential therapeutic targets. Although pharmaceutical agents have been successfully used to block IL-6 in various models, the mechanistic involvement of IL-6 signaling is poorly understood. Utilizing physical exercise as a therapeutic tool could mitigate the deleterious effects of these downstream targets of IL-6 without exacerbating IL-6 levels. However, exercise protocols, such as intensity and duration, are linked to the direct elevation of IL-6 in the muscle and circulation. This may shift the effects of IL-6 toward pathological signaling in the brain, indicating a need for a more comprehensive understanding of exercise protocols related to IL-6 signaling, which could present additional therapeutic opportunities for treating neurological disorders.
